# Induction of Secretagogue Independent Gastric Acid Secretion *via* a Novel Aspirin-Activated Pathway

**DOI:** 10.3389/fphys.2019.01264

**Published:** 2019-10-10

**Authors:** Alice Miriam Kitay, Florentina Sophie Ferstl, Alexander Link, John Peter Geibel

**Affiliations:** ^1^Department of Surgery, School of Medicine, Yale University, New Haven, CT, United States; ^2^Department of Internal Medicine, Medical Faculty University Hospital, Otto-von-Guericke University, Magdeburg, Germany; ^3^Department of Cellular and Molecular Physiology, Yale University School of Medicine, New Haven, CT, United States

**Keywords:** stomach, pH, nitric oxide, reflux, H+, K+ ATPase

## Abstract

Aspirin has been widely recommended for acute and chronic conditions for over 2,000 years. Either single or repetitive doses are commonly used for analgesic and antipyretic reasons and to prevent heart attacks, stroke, and blood clot formation. Recent studies show that it can also be used chronically to dramatically reduce the risk of a variety of cancers. However, prolonged usage of aspirin can cause severe damage to the mucosal barrier, increasing the risk of ulcer formation and GI-bleeding events. In the present study, we show the effects of acute low-dose aspirin exposure as an active secretagogue-inducing gastric acid secretion. Studies were carried out with isolated gastric glands using the pH-sensitive dye BCECF-AM to assess acid secretion. The non-selective NOS inhibitor L-NAME (30 μM), or the specific inhibitor ODQ (1H-[1,2,4]Oxadiazolo[4,3-a]quinoxalin-1-one) was applied while monitoring intracellular pH. The effects of basolateral exposure to aspirin (acetylsalicylic acid, ASA) caused activation of gastric acid secretion *via* the H^+^, K^+^-ATPase. Our data suggest that aspirin increases nitric oxide (NO) production, which in turn activates acid secretion. Exposure of gastric glands to either the non-selective NOS inhibitor L-NAME, and the highly selective, soluble guanylyl cyclase inhibitor 1H-[1,2,4]Oxadiazolo[4,3-a]quinoxalin-1-one (ODQ) effectively inhibited aspirin-dependent gastric acid secretion. Aspirin can be considered as a novel secretagogue, in the way that it activates the H^+^, K^+^-ATPase. With increased daily aspirin consumption, our findings have important implications for all individuals consuming aspirin even in low doses and the potential risks for increased acid secretion.

## Introduction

Salicylate use as a medicinal agent dates back to antiquity, and to this date, aspirin (acetylsalicylic acid, ASA) remains one of the most frequently used pharmaceuticals worldwide (Baron et al., 2013). In the late 1800s, German Chemist Felix Hoffmann was credited with the synthesis of aspirin (Whitlock et al., 2015), which propelled its use for a variety of maladies. To date, its importance in medical therapeutic approaches continues to increase (Rose et al., 2011; Rothwell et al., 2011). Aspirin’s main use has been for fever, pain, and inflammation reduction (Vane and Botting, 1997). Recently, aspirin has been shown to have effects as a low-dose therapeutic for primary and secondary prevention of cardiovascular diseases (Baron et al., 2013). Aspirin demonstrated a significant reduction in non-fatal myocardial infarction (MI) and transient ischemic attack by approximately 50% (Serebruany et al., 2005; Schror, 2007); due to these astonishing results, it is now recommended prophylactilly for all members of the population over 40. More recently, aspirin has been shown to have a chemoprotective effect for colorectal cancer, when taken at low dose for five or more years (Lanas et al., 2011). Outcomes from these studies have shown at least a 27% reduction in risk of colorectal cancer (Schror, 2011). Furthermore, studies now show that similar low-dose aspirin consumption for extended periods can also reduce risk of melanoma (Joosse et al., 2009; Zhu et al., 2015), and also pancreatic cancer (Risch et al., 2017). These protective effects have been suggested to be *via* the inhibition of the COX enzymes, which further reduces inflammatory and immune responses, and is one of the main mechanisms involved in decreasing tumor risk of colonic malignancies (Lanas et al., 2011). The effect of aspirin varies by dose: at low dosage (<100 mg per day), aspirin affects the platelets by the irreversible inhibition of the cyclooxygenases type 1 (COX1), ultimately leading to an inhibition of thromboxane A (TXA) (Hull, 2005); at higher dosage (650 mg–8 g per day), aspirin fulfills characteristics of an analgesic, antipyretic, and anti-inflammatory by inhibiting COX1 and COX2, which leads to a blockage of prostaglandin synthesis (Baron et al., 2013).

Despite all of these advantages, aspirin has in certain cases been shown to have severe adverse effects such as abdominal pain, dyspepsia, nausea, vomiting, and allergies (Baron et al., 2013). In the stomach, aspirin can be responsible for severe damage to the mucosa within the GI tract, leading to an increased risk of ulcer formation (Serebruany et al., 2005; Kitay et al., 2018) and raising the number of major gastric bleeding events by more than 50% in patients taking low-dose aspirin (Lanas et al., 2011). The inhibition of prostaglandin synthesis has been thought to be the primary reason for these gastrointestinal adverse effects (Shim and Kim, 2016). Aspirin has the ability to bind and acetylate prostaglandinendoperoxide synthase 1 (PTGS1 = COX1) and 2 (PTGS2 = COX2) irreversibly (Hull, 2005; Schror, 2011). These enzymes convert arachidonic acid into prostaglandins, eicosanoids, and prostacyclin (Ganjehei and Becker, 2015). This affects and reduces the mucus-buffers-phospholipid layer. Thus, regular aspirin intake interferes with the generation of protective factors necessary for maintaining a barrier to gastric acid (Yandrapu and Sarosiek, 2015).

**Figure 1:**
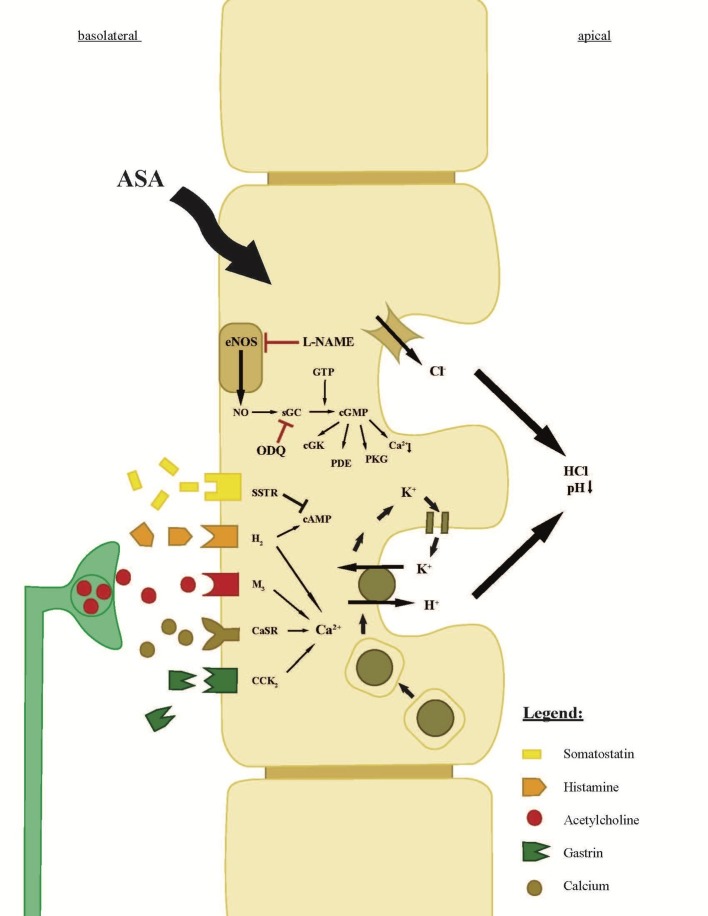
Scheme of the parietal cell of a gastric gland. The scheme explains receptors, ion transporters, and the neuroendocrine regulation of gastric acid secretion (Kitay et al., 2017). The components of concentrated hydrochloric acid are secreted by proton pumps (H^+^, K^+^ ATPases) and chloride channels. Aspirin enters the parietal cell from the bloodside (basolateral) and leads to an intracellular increase of the signaling molecule NO, converted by NOS (Premaratne et al., 2001; Berg et al., 2004; Wei et al., 2018). The increase of NO leads to an increase of sGC, which increases intracellular cGMP levels and ultimately leads to the insertion of the H^+^, K^+^ ATPase on the apical membrane of the parietal cell. The conversion of NO can be inhibited by L-NAME, an analog of arginine, which inhibits NO production. Moreover, the intracellular NO/sGC/cGMP pathway can be inhibited by the sGC-inhibitor ODQ. Intracellular rise in cAMP (by histamine) or Ca^2+^ (by histamine, acetylcholine, Ca^2+^, or gastrin) following the stimulation of the basolateral receptors has a similar effect on the H^+^, K^+^ ATPases like cGMP. The direct neuronal stimulation appears by acetylcholine from the ENS, whereas the hormonal regulation of the parietal cell is dependent on neighboring, histamine-secreting ECL-cells and on G-cells, which secrete gastrin. Gastrin also influences histamine secretion in ECL-cells and leads to an increase of histamine secretion, which is described as the “histamine-gastrin-axis.” Somatostatin, secreted by D-cells, decreases intracellular cAMP levels and will stop the insertion and activation of H^+^, K^+^ ATPases.

The regulation of gastric acid secretion is operated by neuronal, hormonal, and endocrine stimuli affecting the gastric glands of the stomach (Kopic and Geibel, 2013) (Figure 1). The parietal cell is the main player in gastric acid secretion, excreting hydrochloric acid into the lumen by activation of the H^+^, K^+^-ATPase, and various chloride channels, e.g. CFTR, located on the apical surface of the parietal cell (Figure 1; Kopic and Geibel, 2010). Proton pump inhibitors (PPIs), such as omeprazole, serve as the common first line therapy for gastric hypersecretion and acid-related diseases by targeting the H^+^, K^+^-ATPase (Kirchhoff et al., 2011). Recently, we identified an additional apical proton extrusion pathway: the vacuolar H^+^-ATPase that is not blocked by PPI therapy (Kopic et al., 2012).

In this study, we investigated the direct effect of aspirin on the parietal cell and its activation of the H^+^, K^+^-ATPase. We show that aspirin can stimulate the parietal cell and the H^+^, K^+^-ATPase to secrete acid in the absence of known secretagogues. Furthermore, we demonstrate that this aspirin-induced process is *via* activation of NO synthesis within the parietal cell. When either the non-selective NOS inhibitor L-NAME (*N*_ω_-Nitro-L-arginine methyl ester hydrochloride) or the specific sGC inhibitor ODQ was added, the aspirin-induced acid secretion was abolished. In fact, NO signaling is found to be an important pathway in many processes within the cells of the human body (Geller and Billiar, 1998; Sanhueza et al., 2016). Activation of the intracellular receptor NO-GC through NO increases the soluble guanylyl cyclase (sGC) and further to accumulation of cyclic guanosine monophosphate (cGMP) (Ignarro et al., 1987; Koesling et al., 2016; Kitay et al., 2017). We hypothesize that aspirin-stimulated gastric acid secretion in parietal cells occurs through the NO/NO-GC/cGMP signaling pathway, which can be inhibited by the non-selective NOS inhibitor L-NAME or the sGC/cGMP-dependent specific inhibitor ODQ.

## Materials and Methods

### Tissue Preparation

Tissue was obtained from male Sprague-Dawley rats with a weight of 250–400 g (Charles River Laboratory), housed in climate and humidity-controlled light-cycled rooms, fed standard chow with free access to water 24 h. Animals were handled according to the humane practices of Animal Care established by the Institutional Animal Care and Use Committee at Yale University. Ethical consent was ensured by the IACUC Approval 2015–07654. Prior to experiments, animals were food deprived for 12–16 h with free access to water. Animals were euthanized with an overdose of isoflurane. Laparotomy and the removal of the stomach were performed. The corpus of the stomach was separated with a longitudinal incision, washed with ice-cold HEPES to remove residual food particles and sliced into 0.2-cm square sections.

### Isolation of Gastric Glands

After the corpus was excised and transferred to the stage of a dissecting microscope, individual glands were isolated using the hand-dissection technique as described previously (Waisbren et al., 1994; Geibel et al., 2001). Gastric glands were allowed to adhere to coverslips coated with 0.5 μl of the biological adhesive CellTak (Collaborative Research, MA, USA). The coverslip was attached to a perfusion chamber as previously outlined (Kopic et al., 2010).

### Digital Imaging for Intracellular pH Measurements

The isolated gastric glands were incubated in a room tempered HEPES-buffered Ringer’s solution containing 10 μM of the pH-sensitive dye BCECF-AM(2′,7′)-bis-(2-carboxyethyl)-5-(and-6)-carboxy-flourescin-acetomethylester (Santa Cruz Biotechnology, TX, USA) for 20 min. Following this, the chamber was placed on the stage of an inverted microscope (Olympus IX50) and flushed with a HEPES-buffered Ringer’s solution for at least 5 min to remove residual non-deesterified dye. The chamber was thermostatically controlled at 37°C. The epifluorescence mode with an ×40 objective was used and the BCECF excited at the wavelengths of 490 ± 10 nm and 440 ± 10 nm. Between 6 and 14 regions of interest were outlined per gland. During the experimental cycle, the fluorescent signal was monitored at 530 ± 10 nm every 10 s with the help of an intensified charge-coupled device camera. Individual images and intensity values were recorded along with the emission data showing the 440/490 ratio for real-time intensity measurements (Kopic et al., 2012; Ferstl et al., 2016). At the end of the cycle, cells were calibrated with the High K^+^/Nigericin calibration technique to calculate the pH from the emission data as described in previous studies (Boron et al., 1994).

The recovery of intracellular pH (pH_i_) demonstrates the rate of proton extrusion by individual parietal cells after acid/proton loading using the NH_4_Cl prepulse technique (Kopic et al., 2012). Therefore, the cells were superfused with a HEPES-buffered solution containing 40 mM NH_4_Cl. Parietal cells were subsequently flushed with 0Na^+^ solution to eliminate the Na-H-Exchanger (NHE) and trapping protons within the cytosol causing a strong drop of pH. Cells were then flushed with HEPES and finally with the High K^+^ solution. Recovery rates are expressed as ΔpH/min.

### Chemicals and Reagents

All solutions were adjusted to a temperature of 37°C and a pH of 7.4, with the exception of High K^+^ calibration solution, which was titrated to 7.0. The final osmolality of all solutions was 300 mOsm. All chemicals, including Omeprazole, Carbachol, Aspirin, L-NAME, diethylamine NONOate sodium salt hydrate, and ODQ were obtained from Sigma Aldrich (St. Louis, MO, USA) and J.T. Baker (Phillipsburg, NJ, USA).

### Statistical Analysis

Data were checked for outliers *via* the ESD method. The unpaired Student’s *t*-test and the non-parametric, one way ANOVA-test were performed using the Graphpad/Prism software to analyze differences in pH_i_ recovery rates. ΔpH/min values are presented as mean ± SEM. All results with *p* < 0.05 were considered significant.

## Results

**Figure 2:**
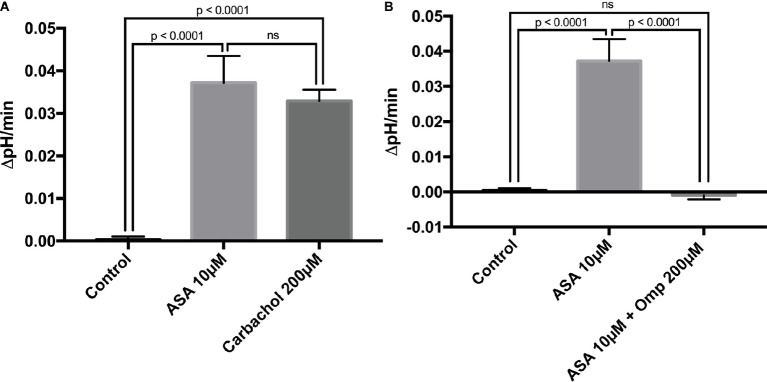
**(A)** Low-dose aspirin causes gastric acid secretion. Bar graph summarizes the effect of aspirin at 10 μM on resting parietal cells (0.0372 ± 0.00629 ΔpH/min) (*n* = 40). The classical secretagogue carbachol at 200 μM resulted in a comparable rate of pH_i_ recovery (0.03291 ± 0.002645 ΔpH/min) (*n* = 168). Isolated gastric glands of rats were loaded with the pH-sensitive dye BCECF and excited at 490 ± 10 and 440 ± 10 nm. The recovery rate of pH_i_ was calculated from the slope in the absence of Na^+^ after acid loading, using NH_4_Cl prepulse technique. The control shows a low basal proton efflux of the resting parietal cell in the absence of stimulatory agents (0.0003711 ± 0.0006969 ΔpHi/min) (*n* = 66). **(B)** Aspirin-induced gastric acid secretion works *via* the H^+^, K^+^-ATPase. After the application of the PPI omeprazole at 200 μM, results demonstrate a low basal proton efflux of the resting parietal cell in the presence of the stimulatory agent aspirin at 10 μM (−0.000905 ± 0.00121 ΔpHi/min) (*n* = 27).

**Figure 3:**
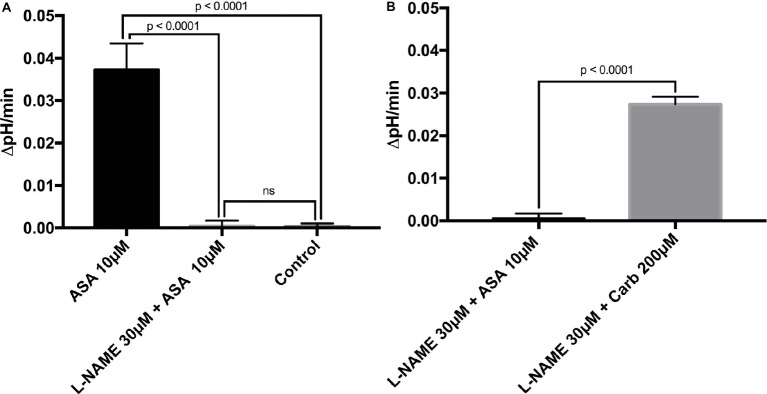
**(A)** L-NAME can inhibit aspirin-induced gastric acid secretion. After adding the non-selective NOS inhibitor L-NAME at 30 μM to our solutions, results demonstrate a low basal proton efflux of the resting parietal cell in the presence of the stimulatory agent aspirin (0.0004409 ± 0.001308 ΔpH/min) (*n* = 85). Thus, L-NAME is a potent and significant inhibitor of aspirin-dependent gastric acid secretion (0.0004409 ± 0.001308 vs. 0.0372 ± 0.00629 ΔpH/min, *p* < 0.0001). **(B)** Carbachol stimulates the parietal cell to secrete gastric acid in the presence of the NOS inhibitor L-NAME. The effect of the classical secretagogue carbachol at 200 μM on rat gastric glands cannot be abolished by the application of the NOS inhibitor L-NAME 30 μM (0.02736 ± 0.001776 ΔpH/min) (*n* = 123). We conclude that aspirin and carbachol differ in the intracellular pathway of the assembly and activation of the H^+^, K^+^-ATPase. Carbachol works through the muscarinic receptor and increases intracellular Ca^2+^ levels, whereas aspirin induces gastric acid secretion through NO.

**Figure 4:**
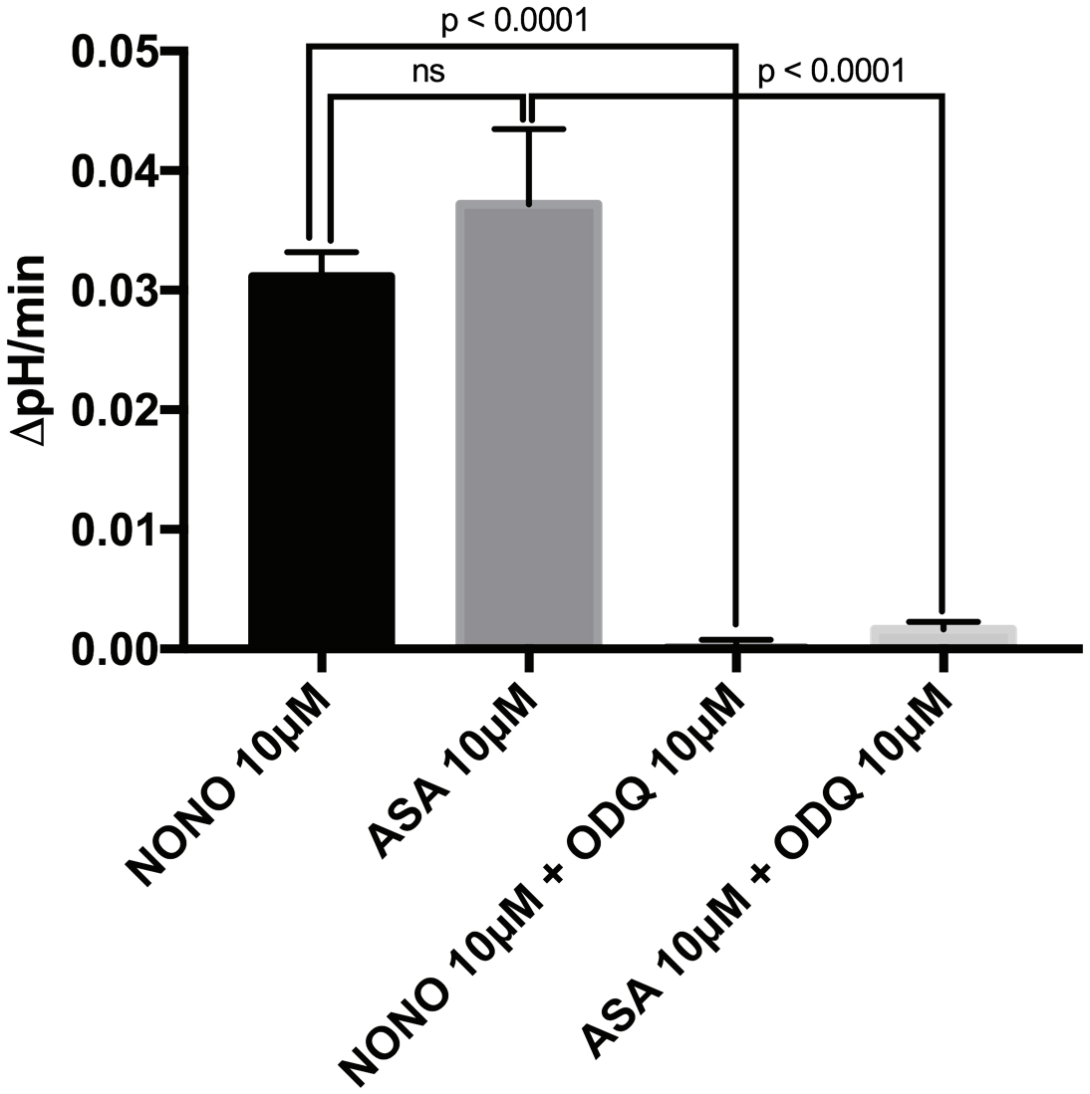
Bar graph shows the effect of the direct NO-donor diethylamine NONOate sodium salt hydrate (NONO) at 10 μM and of the sGC-inhibitor ODQ. The application of the NONOate at 10 μM to our solutions resulted in a significant increase of gastric acid secretion (0.03115 ± 0.002053 ΔpH/min) (*n* = 182). By adding the sGC-inhibitor ODQ at 10 μM to the 10 μM NONOate-containing solutions, we were able to abolish this effect completely to the level of basal proton efflux (0.00009813 ± 0.0006929 ΔpH/min) (*n* = 140). Furthermore, a combination of 10 μM aspirin-containing solutions with ODQ at 10 μM resulted in a significantly lower rate than aspirin without ODQ [0.001641 ± 0.0006249 ΔpH/min (*n* = 89) vs. 0.0372 ± 0.00629 ΔpH/min (*n* = 40), *p* < 0.0001]. With the application of the direct NO-donor diethylamine NONOate sodium salt hydrate and the selective sGC inhibitor ODQ, we prove that aspirin-stimulated gastric acid secretion is working *via* the intracellular NO/sGC/cGMP pathway.

To investigate the direct cellular effect of aspirin on parietal cell acid secretion, we undertook studies using fasted rats with *ad lib* access to fluids (Ferstl et al., 2016). To assay acid secretion, we used the pH-sensitive dye BCECF to give semi-real time measurements of proton extrusion in glands exposed to aspirin. A very low to negligible basal rate of pH_i_ recovery was observed in the absence of secretagogues (0.0003711 ± 0.0006969 ΔpH/min) (*n* = 66) (Figure 2A). Superfusing the glands with solutions containing low-dose aspirin (10 μM) induced a stimulatory effect of aspirin-sensitive proton extrusion in the absence of secretagogues resulting in a significant pH_i_ recovery rate (0.0372 ± 0.00629 ΔpH/min) (*n* = 40) (Figure 2A). The aspirin pH_i_ recovery rate showed a highly significant difference in ΔpH/min compared to non-stimulated conditions, (*p* < 0.0001) (Figure 2A). In a separate series, we added the classical secretagogue carbachol (200 μM) to the glands, which induced a robust dose-dependent proton secretion with a significant increase of pH_i_ recovery rate (0.03291 ± 0.002645 ΔpH/min) (*n* = 168) (Figure 2A). We compared the aspirin-sensitive secretagogue rate to the classical secretagogue carbachol and found comparable rates of proton secretion showing the potency of low-dose aspirin (Figure 2A). To determine if the aspirin-dependent secretagogue stimulation of acid secretion is omeprazole sensitive, we conducted studies combining aspirin exposure with the PPI omeprazole (200 μM). Adding omeprazole (200 μM) to the low-dose aspirin (10 μM) containing superfusion bath induced a significant inhibition of proton extrusion (−0.000905 ± 0.00121 ΔpH/min) (*n* = 27) (Figure 2B) to a level comparable to non-stimulated resting controls (0.0003711 ± 0.0006969 ΔpH/min) (*n* = 66) (Figure 2B) suggesting that the majority of the aspirin effect was *via* the H^+^, K^+^-ATPase. Unsurprisingly, there was no significant difference between the control and the combination of aspirin + omeprazole (*p* = ns), which confirmed that the majority of the effect is *via* the H^+^, K^+^-ATPase (Figure 2B). To investigate the intracellular pathway for aspirin activation of the parietal cell, we added the non-selective NOS inhibitor L-NAME (30 μM) to our solutions. Our results demonstrate a significant decrease of gastric acid secretion in the presence of L-NAME compared to aspirin-induced gastric acid secretion [0.0372 ± 0.00629 ΔpH/min (*n* = 40) vs. 0.0004409 ± 0.001308 ΔpH/min (*n* = 85), *p* < 0.0001] (Figure 3A). In a different series of experiments, we examined the potency of L-NAME to inhibit carbachol-induced gastric acid secretion. Results of these studies show that this activation is not NO sensitive [0.02736 ± 0.001776 ΔpH/min (n = 123) vs. 0.0004409 ± 0.001308 (*n* = 85), *p* < 0.0001] (Figure 3B). Following these studies, we used diethylamine NONOate sodium salt hydrate (1,1-diethyl-2-hydroxy-2-nitroso-hydrazine sodium) (10 μM), a selective NO activator, to confirm the specificity of the novel aspirin finding, that gastric acid secretion can be activated by the intracellular NO pathway. We showed a significant stimulation of the parietal cell acid secretory pathway by the NO donor (0.03115 ± 0.002053 ΔpH/min) (*n* = 182) (Figure 4). We then used the specific sGC inhibitor ODQ to determine if aspirin activated this part of the NO pathway. These studies shown in Figure 4 demonstrate that aspirin indeed works *via* the NO/sGC/cGMP pathway (0.001641 ± 0.0006249 ΔpH/min) (*n* = 89).

We conclude that the novel effect of aspirin causing gastric acid secretion occurs *via* NO signaling and activation of the NO-GC, which further leads to an intracellular increase in the downstream effectors sGC and cGMP. These studies for the first time elucidate a novel effect of aspirin on gastric acid secretion that targets the H^+^, K^+^-ATPase. This activation occurs in the absence of secretagogues suggesting that prior to beginning an aspirin regiment using either normal low-dose aspirin, or enteric coated aspirin, it will be important to use an acid suppression therapy to prevent potential erosions.

## Discussion

For over 2,000 years, aspirin (willow root) has been used to treat a variety of conditions (Jones, 2011). More recently, it has been used to treat everything from chronic pain to prevention of heart attacks, strokes, and clot formation (Whitlock et al., 2015). Now, there are substantial reports that aspirin can reduce the risk of a variety of cancers when taken on a daily basis for extended periods of time (Rothwell et al., 2011). With this further increase in aspiring use comes the potential for increases in patients suffering from gastritis (Baron et al., 2013).

Historically, it has been shown that aspirin leads to modulations in the level of mucous production and that this reduction in mucous may be responsible for the increase in erosions of the lining and the associated gastric bleeds (Lanas et al., 2011).

In the present study, we wished to address another potential adverse effect of aspirin namely that it acts as a secretagogue and leads to acid secretion from the gastric glands.

Using an isolated gastric gland preparation, we examined the effects of aspirin delivered to the basolateral membrane of the glands (blood side). When 10 μM aspirin was added to the basolateral perfusate, we observed a significant increase in acid secretion suggesting that as aspirin levels rise in the blood stream they can induce acid secretion even in the absence of the classical secretagogues.

To examine the mechanism, we looked at the NOS pathway as aspirin is known to cause an upregulation in NO production that would lead to activation of the gastric H^+^, K^+^-ATPase (See Figure 3). This hypothesis was further confirmed in the studies shown in Figures 3 and 4 where we employed the NOS inhibitors L-NAME and ODQ (a specific NOS inhibitor). In both cases, there was a complete cessation in aspirin-induced acid secretion.

Our study now shows that should a patient be considered “aspirin sensitive” in that they have acute gastritis that suggesting an enteric aspirin may no longer be sufficient to protect from gastric erosion. We conclude that elevations of aspirin in the bloodstream can lead to acid secretion as aspirin can directly activate the parietal cells *via* the NO pathway. For these reasons, patients wishing to have the beneficial effects of aspirin should consider the inclusion of agents to block acid secretion.

## Ethics Statement

This study was carried out in accordance with the recommendations of Institutional Animal Care and Use Committee at Yale University. The protocol was approved by the Institutional Animal Care and Use Committee at Yale University.

## Author Contributions

AK was responsible for conducting the experiments, discussing the data, writing the manuscript, constructing the figures, interpretation of the data, and statistical analysis. FF was responsible for conducting the experiments, discussing the data, and statistical analysis. AL was responsible for constructive review of the final manuscript. JG was responsible for experimental design, obtaining funding for the study, interpretation of the data, and review of the final manuscript.

### Conflict of Interest

The authors declare that the research was conducted in the absence of any commercial or financial relationships that could be construed as a potential conflict of interest.

## References

[ref1] Baron J. A. Senn S. Voelker M. Lanas A. Laurora I. Thielemann W.. (2013). Gastrointestinal adverse effects of short-term aspirin use: a meta-analysis of published randomized controlled trials. Drugs R D 13, 9–16. 10.1007/s40268-013-0011-y, PMID: 23532576 PMC3627011

[ref2] Berg A. Redeen S. Ericson A. C. Sjostrand S. E. (2004). Nitric oxide - an endogenous inhibitor of gastric acid secretion in isolated human gastric glands. BMC Gastroenterol. 4:16. 10.1186/1471-230X-4-16, PMID: 15298720 PMC514546

[ref3] Boron W. F. Waisbren S. J. Modlin I. M. Geibel J. P. (1994). Unique permeability barrier of the apical surface of parietal and chief cells in isolated perfused gastric glands. J. Exp. Biol. 196, 347–360. PMID: 7823033 10.1242/jeb.196.1.347

[ref4] Ferstl F. S. Kitay A. M. Trattnig R. M. Alsaihati A. Geibel J. P. (2016). Secretagogue-dependent and -independent transport of zinc hydration forms in rat parietal cells. Pflugers Arch. 468, 1877–1883. 10.1007/s00424-016-1889-3, PMID: 27757581

[ref5] Ganjehei L. Becker R. C. (2015). Aspirin dosing in cardiovascular disease prevention and management: an update. J. Thromb. Thrombolysis 40, 499–511. 10.1007/s11239-015-1267-6, PMID: 26323750

[ref6] Geibel J. P. Wagner C. A. Caroppo R. Qureshi I. Gloeckner J. Manuelidis L.. (2001). The stomach divalent ion-sensing receptor scar is a modulator of gastric acid secretion. J. Biol. Chem. 276, 39549–39552. 10.1074/jbc.M107315200, PMID: 11507103

[ref7] Geller D. A. Billiar T. R. (1998). Molecular biology of nitric oxide synthases. Cancer Metastasis Rev. 17, 7–23. 10.1023/A:1005940202801, PMID: 9544420

[ref8] Hull M. A. (2005). Cyclooxygenase-2: how good is it as a target for cancer chemoprevention? Eur. J. Cancer 41, 1854–1863. 10.1016/j.ejca.2005.04.013, PMID: 16002278

[ref9] Ignarro L. J. Buga G. M. Wood K. S. Byrns R. E. Chaudhuri G. (1987). Endothelium-derived relaxing factor produced and released from artery and vein is nitric oxide. Proc. Natl. Acad. Sci. USA 84, 9265–9269. 2827174 10.1073/pnas.84.24.9265 PMC299734

[ref10] Jones A. W. (2011). Early drug discovery and the rise of pharmaceutical chemistry. Drug Test. Anal. 3, 337–344. 10.1002/dta.301, PMID: 21698778

[ref11] Joosse A. Koomen E. R. Casparie M. K. Herings R. M. Guchelaar H. J. Nijsten T. (2009). Non-steroidal anti-inflammatory drugs and melanoma risk: large Dutch population-based case-control study. J. Invest. Dermatol. 129, 2620–2627. 10.1038/jid.2009.201, PMID: 19587697

[ref12] Kirchhoff P. Socrates T. Sidani S. Duffy A. Breidthardt T. Grob C.. (2011). Zinc salts provide a novel, prolonged and rapid inhibition of gastric acid secretion. Am. J. Gastroenterol. 106, 62–70. 10.1038/ajg.2010.327, PMID: 20736941

[ref13] Kitay A. M. Link A. Geibel J. P. (2017). Activation of secretagogue independent gastric acid secretion via endothelial nitric oxide synthase stimulation in rats. Cell. Physiol. Biochem. 44, 1606–1615. 10.1159/000485755, PMID: 29212068

[ref14] Kitay A. M. Schneebacher M. T. Schmitt A. Heschl K. Kopic S. Alfadda T.. (2018). Modulations in extracellular calcium lead to H(+)-ATPase-dependent acid secretion: a clarification of PPI failure. Am. J. Physiol. Gastrointest. Liver Physiol. 315, G36–G42. 10.1152/ajpgi.00132.2017, PMID: 29517927

[ref15] Koesling D. Mergia E. Russwurm M. (2016). Physiological functions of NO-sensitive guanylyl cyclase isoforms. Curr. Med. Chem. 23, 2653–2665. 10.2174/0929867323666160812145050, PMID: 27776472

[ref16] Kopic S. Corradini S. Sidani S. Murek M. Vardanyan A. Foller M.. (2010). Ethanol inhibits gastric acid secretion in rats through increased AMP-kinase activity. Cell. Physiol. Biochem. 25, 195–202. 10.1159/000276553, PMID: 20110680

[ref17] Kopic S. Geibel J. P. (2010). Update on the mechanisms of gastric acid secretion. Curr. Gastroenterol. Rep. 12, 458–464. 10.1007/s11894-010-0137-9, PMID: 20821079

[ref18] Kopic S. Geibel J. P. (2013). Gastric acid, calcium absorption, and their impact on bone health. Physiol. Rev. 93, 189–268. 10.1152/physrev.00015.2012, PMID: 23303909

[ref19] Kopic S. Wagner M. E. Griessenauer C. Socrates T. Ritter M. Geibel J. P. (2012). Vacuolar-type H+-ATPase-mediated proton transport in the rat parietal cell. Pflugers Arch. 463, 419–427. 10.1007/s00424-011-1060-0, PMID: 22146938

[ref20] Lanas A. Wu P. Medin J. Mills E. J. (2011). Low doses of acetylsalicylic acid increase risk of gastrointestinal bleeding in a meta-analysis. Clin. Gastroenterol. Hepatol. 9, 762–768. e766. 10.1016/j.cgh.2011.05.020 21699808

[ref21] Premaratne S. Xue C. Mccarty J. M. Zaki M. Mccuen R. W. Johns R. A.. (2001). Neuronal nitric oxide synthase: expression in rat parietal cells. Am. J. Physiol. Gastrointest. Liver Physiol. 280, G308–G313. 10.1152/ajpgi.2001.280.2.G308, PMID: 11208555

[ref22] Risch H. A. Lu L. Streicher S. A. Wang J. Zhang W. Ni Q.. (2017). Aspirin use and reduced risk of pancreatic cancer. Cancer Epidemiol. Biomark. Prev. 26, 68–74. 10.1158/1055-9965.EPI-16-0508, PMID: 27999143 PMC5225096

[ref23] Rose P. W. Watson E. K. Jenkins L. S. (2011). Aspirin for prevention of cancer and cardiovascular disease. Br. J. Gen. Pract. 61, 412–415. 10.3399/bjgp11X578133, PMID: 21801534 PMC3103689

[ref24] Rothwell P. M. Fowkes F. G. Belch J. F. Ogawa H. Warlow C. P. Meade T. W. (2011). Effect of daily aspirin on long-term risk of death due to cancer: analysis of individual patient data from randomised trials. Lancet 377, 31–41. 10.1016/S0140-6736(10)62110-1, PMID: 21144578

[ref25] Sanhueza C. Araos J. Naranjo L. Barros E. Subiabre M. Toledo F.. (2016). Nitric oxide and pH modulation in gynaecological cancer. J. Cell. Mol. Med. 20, 2223–2230. 10.1111/jcmm.12921, PMID: 27469435 PMC5134382

[ref26] Schror K. (2007). Aspirin and Reye syndrome: a review of the evidence. Paediatr. Drugs 9, 195–204. 10.2165/00148581-200709030-00008 17523700

[ref27] Schror K. (2011). Pharmacology and cellular/molecular mechanisms of action of aspirin and non-aspirin NSAIDs in colorectal cancer. Best Pract. Res. Clin. Gastroenterol. 25, 473–484. 10.1016/j.bpg.2011.10.016, PMID: 22122764

[ref28] Serebruany V. L. Steinhubl S. R. Berger P. B. Malinin A. I. Baggish J. S. Bhatt D. L.. (2005). Analysis of risk of bleeding complications after different doses of aspirin in 192,036 patients enrolled in 31 randomized controlled trials. Am. J. Cardiol. 95, 1218–1222. 10.1016/j.amjcard.2005.01.049, PMID: 15877994

[ref29] Shim Y. K. Kim N. (2016). Nonsteroidal anti-inflammatory drug and aspirin-induced peptic ulcer disease. Korean J. Gastroenterol. 67, 300–312. 10.4166/kjg.2016.67.6.300, PMID: 27312830

[ref30] Vane J. R. Botting R. M. (1997). Mechanism of action of aspirin-like drugs. Semin. Arthritis Rheum. 26, 2–10. 10.1016/S0049-0172(97)80046-7, PMID: 9219313

[ref31] Waisbren S. J. Geibel J. P. Modlin I. M. Boron W. F. (1994). Unusual permeability properties of gastric gland cells. Nature 368, 332–335. 10.1038/368332a0, PMID: 8127367

[ref32] Wei Q. Korejo N. A. Jiang J. Xu M. Zheng K. Mao D.. (2018). Mitigation of stress from gastric mucosal injuries by mulberry extract may occur via nitric oxide synthase signaling in mice. Tissue Cell 54, 59–64. 10.1016/j.tice.2018.08.007, PMID: 30309511

[ref33] Whitlock E. P. Williams S. B. Burda B. U. Feightner A. Beil T. (2015). “U.S. preventive services task force evidence syntheses, formerly systematic evidence reviews” in Aspirin use in adults: Cancer, all-cause mortality, and harms: A systematic evidence review for the U.S. preventive services task force (Whitlock Rockville, MD: Agency for Healthcare Research and Quality (US)). 26491756

[ref34] Yandrapu H. Sarosiek J. (2015). Protective factors of the gastric and duodenal mucosa: an overview. Curr. Gastroenterol. Rep. 17:24. 10.1007/s11894-015-0452-2, PMID: 26109006

[ref35] Zhu Y. Cheng Y. Luo R. C. Li A. M. (2015). Aspirin for the primary prevention of skin cancer: a meta-analysis. Oncol. Lett. 9, 1073–1080. 10.3892/ol.2015.2853, PMID: 25663859 PMC4314970

