# Supplementary material for: Induction of Secretagogue Independent Gastric Acid Secretion via a Novel Aspirin-Activated Pathway
Source: Front Physiol. 2019 Oct 10;10:1264. doi: 10.3389/fphys.2019.01264 (PMC6795678; doi:10.3389/fphys.2019.01264)
Supplement: Supplementary file 1 [file Data_Sheet_1.PDF]

Tab.1 Solutions

	HEPES	NH_4Cl	$0Na^+$	$0Na^+0K^+$	$HighK^+$
NaCl	117	77	0	0	0
KCl	5	5	5	0	105
NH_4Cl	0	40	0	0	0
$CaCl_2$*7$H_2$0	1	1	1	1	1
$MgSO_4$*7$H_2$0	1.2	1.2	1.2	1.2	1.2
HEPES	32.2	32.2	32.2	32.2	32.2
Glucose	10	10	10	10	0
NMDG	0	0	132.8	137.8	32.8
Mannitol	0	0	0	0	5

Concentrations are expressed in millimolar.
